# Microwave imaging for breast cancer screening: protocol for an open, multicentric, interventional, prospective, non-randomised clinical investigation to evaluate cancer detection capabilities of MammoWave system on an asymptomatic population across multiple European countries

**DOI:** 10.1136/bmjopen-2024-088431

**Published:** 2024-11-02

**Authors:** Daniel Álvarez Sánchez-Bayuela, Juan Fernández Martín, Gianluigi Tiberi, Navid Ghavami, Rubén Giovanetti González, Lina Marcela Cruz Hernánez, Paul Martín Aguilar Angulo, Aarón Darío Martínez Gómez, Ana Rodríguez Sánchez, Alessandra Bigotti, Banafsheh Khalesi, Letizia Pontoriero, Massimo Calabrese, Alberto Stefano Tagliafico, Cristina Romero Castellano

**Affiliations:** 1 Instituto de Investigación Sanitaria de Castilla - La Mancha, Toledo, Spain; 2 University Hospital of Toledo, Servicio de Salud de Castilla - La Mancha, Toledo, Spain; 3 London South Bank University, London, UK; 4 UBT - Umbria Bioengineering Technologies, Perugia, Italy; 5 Fondazione Toscana Life Sciences, Siena, Italy; 6 IRCCS Ospedale Policlinico San Martino, Genova, Italy; 7 University of Genoa, Genova, Italy

**Keywords:** Mass Screening, Breast imaging, Artificial Intelligence

## Abstract

**Introduction:**

Microwave imaging presents several potential advantages including its non-ionising and harmless nature. This open, multicentric, interventional, prospective, non-randomised trial aims to validate MammoWave’s artificial intelligence (AI)-based classification algorithm, leveraging microwave imaging, to achieve a sensitivity exceeding 75% and a specificity exceeding 90% in breast screening.

**Methods and analysis:**

10 000 volunteers undergoing regular mammographic breast cancer screening will be recruited across 9 European centres and invited to participate in the clinical study, involving MammoWave testing on both breasts. MammoWave results will be checked against the reference standard, to be intended as the output of conventional breast examination path (with histological confirmation of cancer cases) with 2 years follow-up. Anonymised clinical and MammoWave’s results, including microwave images, associated features and a label provided by the AI-based classification algorithm, will be collected and stored in a dedicated electronic case report form. The prospective study will involve a comparative analysis between the output of the conventional breast examination path (control intervention) and the labels provided by MammoWave’s AI system (experimental intervention). These labels will categorise breasts into two groups: breast With Suspicious Finding, indicating the presence of a suspicious lesion or No Suspicious Finding, indicating the absence of a lesion or the presence of a low-suspicion lesion. This trial aims to provide evidence regarding the novel MammoWave’s AI system for detecting breast cancer in asymptomatic populations during screening.

**Ethics and dissemination:**

This study was approved by the Research Ethics Committee of the Liguria Region (CET), Italy (CET-Liguria: 524/2023—DB id 13399), the Research Ethics Committee of Complejo Hospitalario de Toledo (CEIC), Spain (CEIC-1094), the National Ethics Committee for Clinical Research (CEIC), Portugal (CEIC-2311KC814), the Bioethical Committee of Pomeranian Medical University in Szczecin, Poland (KB-006/23/2024) and the Zurich Cantonal Ethics Commission, Switzerland (BASEC 2023-D0101). The findings of this study will be disseminated through academic and scientific conferences as well as peer-reviewed journals.

**Trial registration number:**

NCT06291896.

**STRENGTHS AND LIMITATIONS OF THIS STUDY**

- This is the first multicentric, prospective study to evaluate the sensitivity and specificity of MammoWave’s artificial intelligence (AI) solution within the framework of an asymptomatic population-based programme for breast cancer detection.
- The MammoWave device will use an AI-powered clinical decision support system to automatically provide radiologists with a dedicated label indicating the presence or absence of suspicious findings in each breast.
- As a reference standard, the 2-year follow-up with histological confirmation of cancer cases will be used.
- Study limitations include its non-randomised design.
- The axillary region of the breast is not captured within MammoWave’s cup.

## Introduction

 Breast cancer (BC) stands as the most prevalent cancer among women globally, with one in eight women experiencing its impact in their lifetime, rendering it a paramount challenge in public health.^1^,^3^ In response, many countries have instituted national or regional programmes dedicated to early detection, exemplified by screening campaigns aimed at mitigating the burden of BC.^4 5^ At the European level, the European Commission has always been committed to the fight against cancer, stimulating various initiatives such as the Europe’s Beating Cancer Plan and the Mission Cancer, and acting by developing evidence-based recommendations. In fact, the European Commission Initiative on Breast Cancer (ECIBC) regularly updates a list of guidelines to perform and optimise breast screening practices, according to the existing level of evidence in used imaging technologies, age of screening and frequency.^6^,^8^

Full-field digital mammography (DM), also called simply DM and digital breast tomosynthesis (DBT) serve as the gold standard technologies for mammographic screening, having demonstrated efficacy in reducing BC mortality through randomised controlled trials.^59^,^12^ However, these imaging modalities are not without limitations and potential adverse effects, including ionising radiation exposure, breast compression discomfort and inherent performance constraints associated with X-ray-based methods. Notably, dense breast tissue can diminish mammographic sensitivity, leading to reduced detection rates and increased rates of false positives. To mitigate these challenges, recommendations such as double reading by expert radiologists and optimising screening age and frequency have been proposed, considering the risk–benefit ratio of mammography.^7 8 13^ Indeed, risks associated with X-rays’ cumulative effect and low sensitivity in dense breasts, which may be reduced to 57.8%,^14^ limit the use of mammography in younger women or in reduced time intervals. In general, women are eligible for biennial screening only after the age of 45 according to ECIBC; nevertheless, recent studies estimate that 32.4% of new cancers in women aged under 50 are BCs.^3 15^ Moreover, emerging technologies such as Computer Aided Detection systems, based on deep learning algorithms (artificial intelligence, AI), are aiding radiologists in interpretation and decision-making processes, offering potential improvements in screening efficacy.^16^,^22^

Recognising the imperative of early detection, attention has turned towards novel technologies such as microwave breast imaging (MBI) as a promising avenue for overcoming the limitations of conventional X-ray-based approaches. MBI operates without ionising radiation, using radiofrequency (RF) signals to discern between healthy and malignant breast tissues based on their dielectric properties.^23^ Malignant cells, characterised by higher water and sodium accumulation, exhibit distinctive dielectric properties (conductivity and permittivity), allowing for microwave discrimination and cancer detection.^24^ Various MBI prototypes have been developed and clinically tested, among which MammoWave (UBT—Umbria Bioengineering Technologies Srl, Italy) has garnered attention. MammoWave employs low-power microwaves and has demonstrated promising sensitivity for breast lesion detection in previous clinical trials,^25^,^33^ offering a non-compressive and safe imaging alternative that may be suitable for population-based mass BC screening.

Here, we introduce the approved clinical protocol (full title: A Clinical Investigation to Evaluate Microwave Imaging Via MammoWave in a Population-based Screening Program for Early Breast Cancer Detection, v. 1.0 of 04/09/2023) for an open, multicentric, interventional, prospective and non-randomised trial aimed at evaluating, for the first time, MBI via MammoWave within the context of population-based BC screening. The study has been activated in the framework of the European cofunded project MammoScreen (‘Innovative and safe microwave-based imaging technology to make BC screening more accurate, inclusive and female-friendly’), under the programme Horizon Europe 2021–2027, grant ID 101097079. UBT is the sponsor of the study. Potential advantages and current limitations of MammoWave’s solution are reported in box 1.

Box 1 **Use of Mammowave’s artificial intelligence (AI) solution in breast screening: advantages and limitations**

Advantages

- MammoWave’s solution is based on harmless, non-ionising microwave imaging which may allow more frequent examinations with no age restrictions.
- Dedicated facilities and sophisticated settings are not necessary.
- The scanning protocol, lasting 8 min per breast, is simple: a technician will help the patient lie down, introducing the breast in a dedicated cup. Then, microwave images are reconstructed on an external server, and an AI-powered algorithm (machine learning approach) provides a label for each breast.
- MammoWave’s AI is operator-free (no image interpretation is required).

Limitations

- Currently, there are no guidelines including the use of microwave imaging technologies in clinical practice.
- The cost-effectiveness and impact of implementing microwave imaging technologies have not been approached.
- The impact of hormonal changes in scattered radiofrequency signals and corresponding MammoWave microwave images during a woman’s lifetime or menstrual cycle should be investigated.

## Methods and analysis

### MammoWave device and imaging algorithm

**Figure 1:**
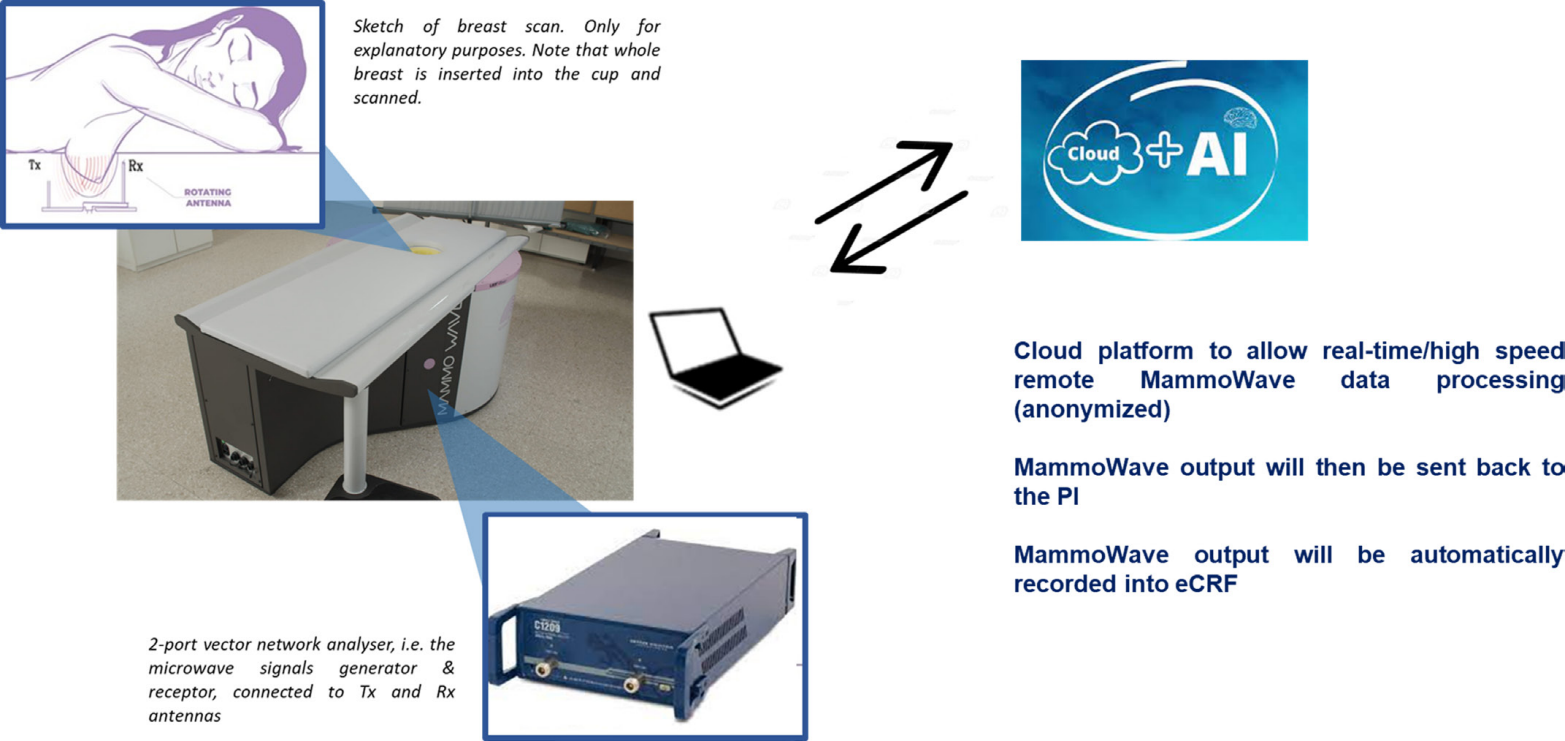
MammoWave system and its operability. AI, artificial intelligence; eCRF, electronic case report form; RX, receiving; TX, transmitting.

MammoWave (figure 1) is a new breast imaging device using safe, non-ionising low-power microwaves (RF signals), akin to those in mobile communications. It consists of two antennas emitting low-power (<1 mW) RF signals in the microwave band (1–9 GHz) to illuminate the breast and measure corresponding scattered electromagnetic fields (denoted as S_21_). The device’s hardware comprises a cylindrical aluminium hub internally coated with an absorption cone structure, creating an anechoic chamber to absorb reflections of electromagnetic waves that could cause noise/artefacts during data acquisition. Two motorised arms support both antennas, rotating around the azimuth to irradiate the breast and capture scattered electromagnetic fields. Both transmitting (TX) and receiving (RX) antennas are connected to a vector network analyser (VNA) for precise signal transmission and measurement. The acquisition configuration involves 10 TX positions grouped into 5 doublet sections with centres at 0°, 72°, 144°, 216° and 288°, with the RX antenna collecting signals at 80 positions around the breast for each TX position. The VNA acquires the S_21_ parameter for 1601 frequencies from 1 to 9 GHz, in 5 MHz increments. A programming logic controller inside the hub controls all components, providing shield from external interferences and structural support. The examination bed, atop the hub, includes a plexiglass cup to accommodate the volunteer’s breast without compression, with various cup sizes available for different breast sizes/types. The examination, lasting 8 min per breast, is performed with the woman lying face down.

MammoWave’s software employs an imaging algorithm based on Huygens’ principle (HP) to generate microwave images, which are intensity maps representing the homogeneity of breast tissues’ dielectric properties.^34 35^ Images are maximum intensity projection coronal two-dimensional (2D) maps of the entire breast volume. Specifically, after acquiring S_21_ data, the HP-based software produces microwave images and parameters describing them, such as the Max/Avg intensity ratio, used to assess images’ non-homogeneity. MammoWave’s output includes a label generated by a novel AI-based classification algorithm trained using previous clinical data (as described in the next subsection).

#### MammoWave’s AI-powered clinical decision support system

MammoWave’s novel software includes an AI (machine learning-based) classification algorithm. This algorithm has been developed using the clinical data collected in a previous international, multicentric study (ClinicalTrials.gov Identifier: NCT04253366). In particular, the labels (reference standard, ie, the output of conventional breast examination path) collected in such study were used for training and testing, with the aim of optimising the AI-based classification algorithm (to be used, prospectively, within this clinical trial).

This AI-based classification algorithm works on the S_21_ data of each scanned breast, which is a matrix of complex electromagnetic signals derived from the measurement performed for multiple positions of the TX and RX antennas and sampled in frequency. Principal component analysis methods were used to reduce the matrix’s dimensionality and fix the number of components so that at least 95% of the variance in the data is accounted for. Various algorithms were tested (support vector machines (SVM), decision trees, random forests, logistic regression, k-nearest neighbours), using the cross-validation methodology and calculating the performances in terms of specificity and sensitivity.^36^ More specifically, the SVM-based algorithm will be used within this trial to categorise breasts into two groups: WSF (breast With Suspicious Finding), indicating the presence of a suspicious lesion, or NSF (No Suspicious Finding), indicating the absence of a lesion or the presence of a low-suspicion lesion. More details related to algorithm’s validation methodology and performances in terms of specificity and sensitivity can be found in Papini *et al*,^36^ where specificity of up to ~90% and sensitivity of up to ~85% have been reported.

**Figure 2:**
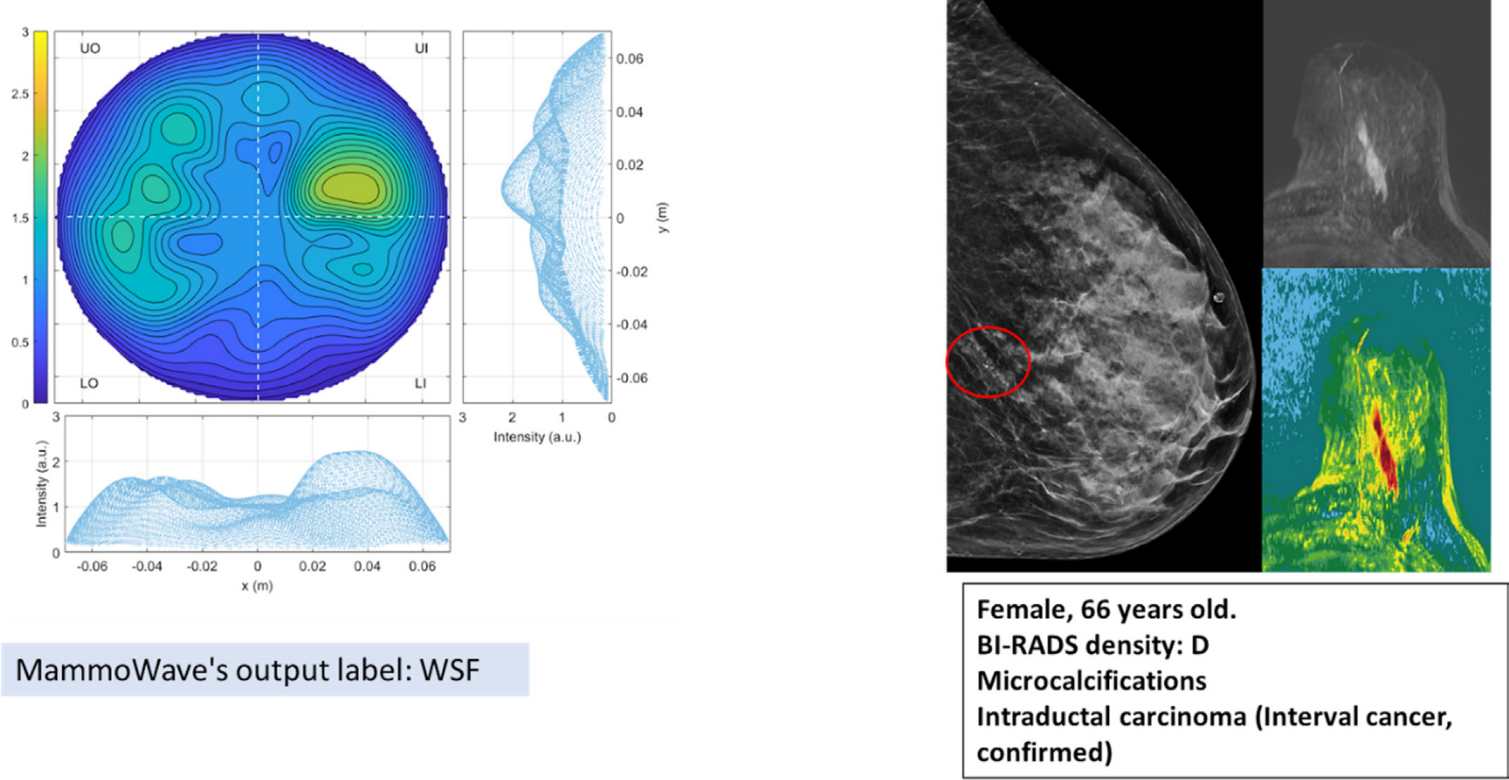
(Left) Example of MammoWave output, which primarily consists of microwave image and includes the label generated by the novel AI-based classification algorithm, WSF for this breast; (Right) reference standard output and details are given for the same breast. AI, artificial intelligence; WSF, with suspicious finding.

As an example, figure 2 shows MammoWave’s output and reference standard output is also shown.

### The clinical study

This study is entitled ‘A multicentric, prospective clinical investigation to evaluate microwave imaging via MammoWave in a population-based screening program for early breast cancer detection to make breast cancer screening more accurate, inclusive, and female-friendly’ and has been activated in March 2024 (ClinicalTrials.gov Identifier: NCT06291896).

#### Hypothesis and objectives

The aim of this clinical study is to validate, prospectively, MammoWave’s AI-based classification algorithm. The hypothesis underlying this clinical study is that MammoWave’s AI-based classification algorithm achieves a sensitivity of over 75% and a specificity of over 90% in the detection of BC within the screening process of asymptomatic individuals currently enrolled in population-based screening programmes.

The primary objective of this study is to generate evidence in the detection of BC by using MammoWave, evaluating its sensitivity and specificity, having as reference standard the output of conventional breast examination path (ie, screening mammogram, integrated with other radiological/histological output when deemed necessary by the responsible Investigator).

The secondary objectives of the study are as follows:

1. Evaluation of the sensitivity of MammoWave in detecting BC according to the American College of Radiology (ACR) BI-RADS classification.^37^
2. Evaluation of the effectiveness of MammoWave versus a conventional reading of digital mammographic images (either 2D or tomosynthesis) and analysis of whether they are equivalent in terms of detection rates of BC (sensitivity),the number of recalls and positive predictive value (for the biopsies performed).
3. Evaluation of the detection rate (sensitivity) of each system according to the type of mammographic radiological finding (microcalcification, mass, asymmetry or architectural distortion group).
4. Evaluation of the impact of breast density (according to the ACR BI-RADS classification^37^ assigned by the radiologist, with or without automatic software if available) on the MammoWave detection rate of BC, number of recalls and positive predictive value (of the biopsies performed).
5. Evaluation of the impact of breast density (according to BI-RADS classification assigned by the radiologist^37^) on the detection rate (sensitivity) of each system depending on the type of finding (microcalcification, mass, asymmetry or architectural distortion group).
6. Comparison of radiological, histological and stage characteristics between the BCs detected by each system.
7. Evaluation of the reproducibility of the AI-based MammoWave software’s results.
8. Evaluation of the improvement in both sensitivity and specificity when retrospectively using AI algorithms to evaluate MammoWave data after its further optimisation with all the data collected in the patients included in the study.
9. Volunteers’ satisfaction related to MammoWave use, through a properly designed questionnaire (given here as online supplemental material), collecting women’s issues during the exam with MammoWave in the language spoken by the women.

Additionally, there is a secondary objective, which is related to MammoWave safety and tolerability assessment. Furthermore, an additional exploratory objective of the clinical study is to establish a predictive model capable of discriminating and classifying lesions in MammoWave outcomes for volunteers with breast lesions, if feasible.

#### Study population, inclusion and exclusion criteria

The study will include consecutive volunteers who are participants of the local, regional or national BC screening programmes. These volunteers will fulfil all the prerequisites and inclusion criteria set forth by such programmes, following the European guidelines on BC screening and diagnosis^15^: asymptomatic women aged between 45 and 74 years, with no indications of BC suspicion and not currently enrolled in other clinical imaging pathways (such as pathological, high-risk or women with implants).

In more detail, participants fulfilling all the following inclusion criteria are eligible for the study: (1) women aged between 45 and 74 years old; (2) asymptomatic; (3) with signed informed consent form collected before starting any study activity; (4) with average risk of BC (every woman except those with known BRCA1, 2, TP53 and/or previous BC); (5) having a radiologist study output obtained using conventional mammographic exams, either 2D DM or 3D DBT (collecting oblique mediolateral projection (MLO) of the right breast, craniocaudal projection (CC) of the right breast, MLO projection of the left breast, CC projection of the left breast), performed within the past month with available results or planned to be performed in the same day of the MammoWave test or the subsequent days and (6) with a spontaneous willingness to comply with the clinical investigation plan (CIP) and recommendations.

The presence of any one of the following criteria will lead to the exclusion of the participant: (1) women with breast prostheses; (2) women with symptoms or some sign of suspected BC; (3) women with known BRCA 1, 2, TP53 or previous BC; (4) pregnant women; (5) women who do not have a mammographic manifestation of the tumour (known occult BC with only axillary manifestation) and (6) women with breast size larger than the largest MammoWave cup size.

#### Study design

**Figure 3:**
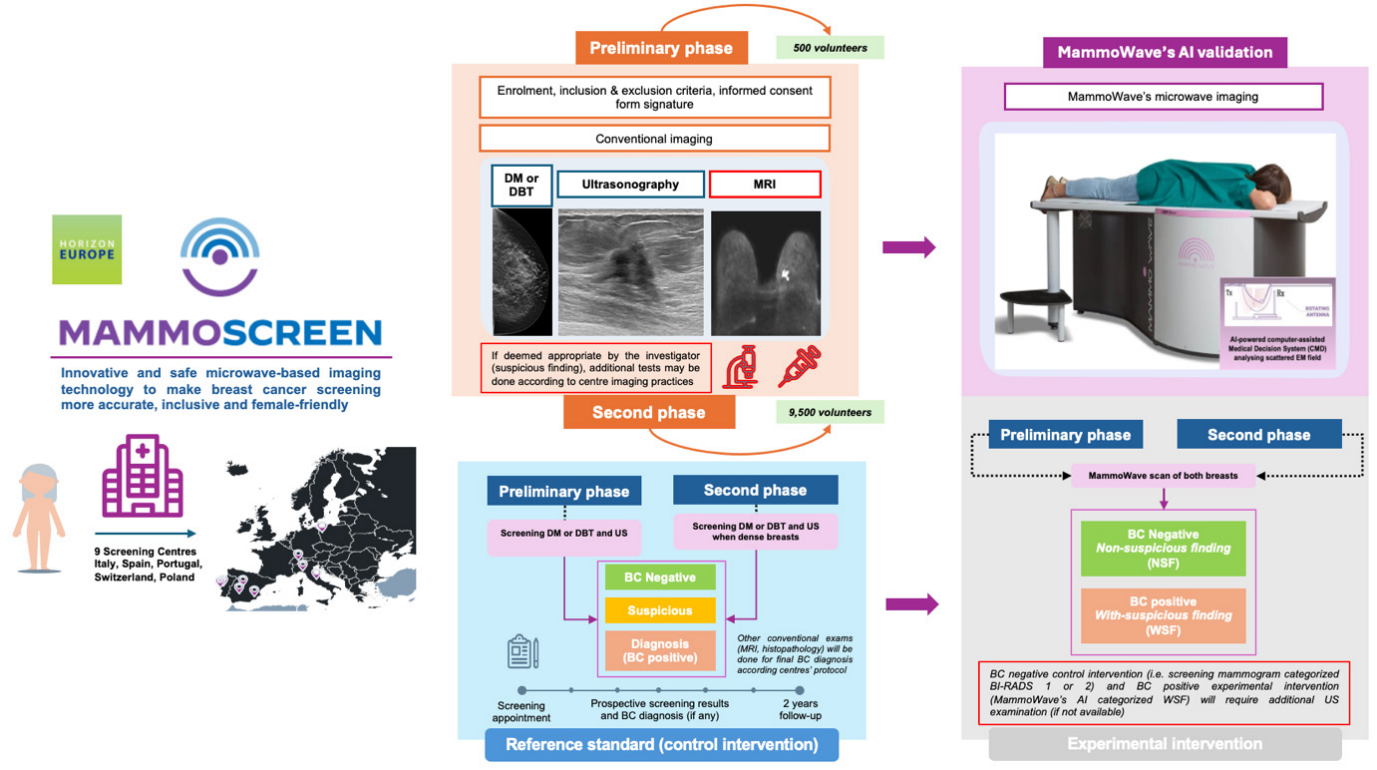
Study design and recruitment process. AI, artificial intelligence; BC, breast cancer; DBT, digital breast tomosynthesis; DM, digital mammography.

This is an open, multicentric, interventional, prospective, non-randomised clinical investigation in which 10 000 volunteers undergoing conventional breast screening examination will also undergo the MammoWave exam. Recruitment of volunteers will be performed at nine centres in different European countries: two Italian hospitals (IRCCS Policlinico San Martino, Genova; and Ospedale San Giovanni Battista—USL Umbria 2, Foligno), one Polish hospital (Pomeranian Medical University Hospital, Szczecin), two Portuguese centres (Champalimaud Foundation, Lisbon and Clínica Dr Passos Ângelo, Lisbon), three Spanish hospitals (Hospital Universitario de Toledo, Toledo; Hospital Universitario Reina Sofía, Córdoba and Hospital General Universitario Morales Meseguer, Murcia) and one Swiss hospital (University Hospital, Zurich). The study (see figure 3) will be composed of two phases:

1. A preliminary phase is planned to validate and fine-tune the optimised MammoWave’s AI classification algorithm. During this phase, the initial cohort will consist of 500 women recruited from all participating clinical sites. In this preliminary stage, all asymptomatic volunteers will undergo conventional mammography and ultrasound (US) examinations at a minimum to ensure optimal diagnostic accuracy. Suspected cases will follow the standard site-specific pathways, which may include additional tests such as MRI and biopsy.
2. During the second phase, the remaining 9500 women will be enrolled. If the MammoWave’s AI label is positive, an US examination will be conducted (in addition to conventional mammography), particularly for women with dense breasts (recall, as per the European Society of Breast Imaging (EUSOBI), recommendations for screening in dense breasts^38^), unless such examination has already been performed. Suspected cases will follow the standard site-specific pathways, which may include additional tests such as MRI and biopsy. After the enrolment of 3000 volunteers, an interim evaluation will be carried out.

Women attending the centres for their scheduled mammogram appointment (or those who have had a mammogram within the past month) will receive information about the study and will be invited to participate on a voluntary base. Women who agree to participate, meet the inclusion and exclusion criteria and sign their informed consent document will undergo the MammoWave examination. All the eligible women will be enrolled consecutively. The reason for non-participating in the study (fear of the new exam, lack of time, lack of information, lost-to-follow-up, breasts size larger than MammoWave’s cup) will be recorded by the investigators.

On completion of MammoWave’s scan of each breast, the data will undergo processing in a cloud platform to enable real-time and high-speed data processing. The output from MammoWave will primarily consist of microwave images (along with parameters that characterise these images) and will include the label generated by the novel AI-based classification algorithm; the label will be then used for final analysis by comparing it with conventional imaging results. Thus, the microwave images (which lack detailed morphological definition) and their associated features will not serve as a basis for diagnostic decisions made by radiologists or investigators.

Regarding intervention, if MammoWave’s results (ie, labels provided by its AI classification system) indicate WSF and there is a disagreement between MammoWave output and conventional mammographic imaging, additional examinations may be warranted. These may include US, if not already conducted, particularly for dense breasts, aligning with EUSOBI recommendations due to the reduced sensitivity of conventional mammography in dense breasts.^38^ All output information, including MammoWave’s results and conventional breast examination findings, will be collected and incorporated into this study. At each site, the investigators will prospectively compare MammoWave labels with the reference standard. Furthermore, on the study’s conclusion, the MammoWave labels will undergo centralised review by blinded specialists who will not have access to the reference standard. This review aims to validate the AI-produced data.

Following the study, a retrospective analysis will be conducted to refine an additional AI-generated algorithm using all acquired data. This algorithm will then be employed to re-evaluate the MammoWave data collected during the study, retrospectively reassessing the endpoints.

Regarding blinding strategies, the study will proceed as follows: first, each mammogram (either full-field DM or DBT) will be independently double-read by two radiologists, with consensus or arbitration for any discordant interpretations, in accordance with European breast screening guidelines (European Guidelines on Breast Cancer Screening and Diagnosis, ECIBC). Following this, all the eligible women will be enrolled consecutively, as detailed above. Blinding will be in place for the participants in the study. The output from MammoWave will be automatically transmitted to the responsible Investigator; blinding will be in place for MammoWave analysis.

#### Study period

Recruitment started in February 2024; recruitment of the last participant is planned for February 2026. The deadline for the first evaluation of the results is planned for December 2026.

#### Choice of comparator/control intervention

MammoWave’s results (labels provided by its AI-based classification algorithm) will undergo validation against a reference standard, which represents the output of the conventional breast examination pathway. This pathway includes screening mammograms conducted with DM or DBT, integrated with other radiological/histological findings as required by the responsible investigator, following the site’s ongoing procedures and clinical circuit. The reference standard’s results will be categorised as ‘positive’ when BC presence is confirmed by histology (ie, BI-RADS 6), otherwise labelled as ‘negative’ (BI-RADS 1, 2 or 3). Specifically, within this study BI-RADS 4 and BI-RADS 5 are only image suspicion criteria: when a biopsy is performed, they will be categorised as ‘positive’ when BC presence is confirmed by histology (ie, BI-RADS 6), otherwise labelled as ‘negative’ (BI-RADS 1, 2 or 3).

Conventional mammogram studies (2D or tomosynthesis) will adhere to approved protocols and clinical practices for breast screening. Radiological reports and interpretations will align with international guidelines, particularly those set by the ACR.^37^ The radiologists involved in this study will have extensive experience in mammographic interpretation, serving as the benchmark during the study. Details such as single or double screening readings and the utilisation of AI-assisted mammographic reading will be documented at each centre. Additionally, the integration of conventional mammogram studies with other radiological/histological findings will occur when deemed necessary by responsible investigators, who will record relevant variables in the electronic case report form (eCRF) following their usual clinical practice.

#### Collected variables

The following clinical variables will be collected per volunteer:

- Age (years).
- Mammographic breast density, rated according to the radiologist’ BI-RADS assessment: A (breasts of fat density), B (breasts with areas of scattered fibroglandular tissue), C (heterogeneously dense breasts), D (extremely dense breasts).
- Breast (right/left) and quadrant (superior/inferior/lateral/medial) where the lesion is located (if present).
- BI-RADS score of the screening mammographic study for each breast (2D or tomosynthesis) according to the degree of suspicion (nominal qualitative variable).
- Breast type assigned by MammoWave (nominal qualitative variable): WSF, NSF.
- Radiological findings in the mammographic readings made by radiologists (none, nodule, microcalcifications, distortion or asymmetric density).
- Additional tests (US, MRI) performed, with correspondent outcomes (BI-RADS score, confirmed presence of BC, not conclusive, confirmed absence of BC).
- Biopsy performed, if any, with outcomes. When a biopsy is performed, the kind of method (fine-needle aspiration biopsy or core needle biopsy), histology of the lesion and immunohistochemistry are recorded as:
  - Histopathological evaluation of BC according to typology (infiltrating ductal carcinoma, infiltrating lobular carcinoma, ductal carcinoma in situ and other types).
  - Histopathological evaluation of BC according to tumour grade (I, II, III).
  - Molecular evaluation of BC (luminal A, luminal B, HER-2 positive, triple negative).
- Size of the confirmed lesion of BC (tumour), according to the tumour, node and metastases (TNM) staging system^39^ (T0: no evidence of primary tumour; T1mi: tumour less than or equal to 1 mm; T1a: tumour between 1 and 5 mm (included); T1b: tumour between 5 mm and 1 cm (included); T1c: tumour between 1 and 2 cm (included); T2: tumour between 2 and 5 cm (included); T3: tumour larger than 5 cm; T4: tumour of any size with involvement in skin, chest wall or inflammatory BC).
- Lymph node involvement of BC, according to TNM staging system (N0: no evidence of BC in lymph nodes or areas of BC less than 0.2 mm; N1: spread of BC in 1–3 internal axillary or mammary nodes; N2: spread of BC in 4–9 axillary nodes/spread of BC in internal mammary nodes, but not in axillary; N3: spread of BC to 10 or more nodes/spread of BC to supraclavicular or infraclavicular nodes).
- BC stage according to TNM staging system (0, IA, IIA, IIB, IIIB, IIIC).
- Suggested surgical treatment (conservative (quadrantectomy/lumpectomy); radical).
- Women’s satisfaction (a properly designed questionnaire collecting women’s issues and plus during the exam with MammoWave in the language spoken by the women).
- Adverse events (AEs).

#### Study outcomes

The primary outcomes will be measured as follows:

- MammoWave’s sensitivity (percentage of ‘true positive’ results) while using an optimised AI-based classification algorithm.
- MammoWave specificity (percentage of ‘true negative’ results) while using an optimised AI-based classification algorithm.

The secondary outcomes will be measured as follows:

- BC detection rates of each system by type of lesion (microcalcification, mass, asymmetry or architectural distortion group).
- BC detection rates, recall and positive predictive value (biopsy) of each system, also by breast density (types A and B being low-density breasts (fat), C and D high-density breasts (high content of fibroglandular tissue)).
- BC detection rates of each system by BI-RADS classification.^37^
- BC detection rates of each system by type of lesion (microcalcification, mass, asymmetry or architectural distortion group) and density (fat or high content of fibroglandular tissue).
- Programme screening rate, calculated as the proportion of women diagnosed with BC in the study population.
- BC detection rates of each system by histological type and size of the BC and BI-RADS classification.^37^
- MammoWave’s AI classification algorithm’s sensitivity (frequency of ‘true positive’ results) and specificity (frequency of ‘true negative’ results) after a retrospective adjustment using the data at the end of the study.
- BC detection rate, recalls and positive predictive value (biopsy), also by breast density (types A and B or C and D) after retrospective AI adjustment using the data at the end of the study.
- Programme screening rate, calculated as the proportion of women diagnosed with BC in the study population after a retrospective adjustment using the data at the end of the study.
- Evaluation of the reproducibility of results comparing the lectures performed at the study centres and the centralised lectures.
- Volunteers’ satisfaction through a properly designed questionnaire collecting women’s issues during the exam with MammoWave in the language spoken by the women.

Regarding safety outcomes, all the events will be registered, and they will be identified as related or not to MammoWave.

#### Follow-up

The trial design will allow the comparative evaluation of both the conventional mammogram and MammoWave’s AI solution in terms of cancer detection rate, the number of recalls and positive predictive value, having as reference standard the 2-year follow-up of each volunteer. This follow-up allows to check retrospectively the possible rate of interval BC, and if this was reported as a false positive by MammoWave.

#### Study’s ethical conduct

The study will be carried out following the protocol and with principles enunciated in the current version of the Declaration of Helsinki, the guidelines of Good Clinical Practice issued by the International Council for Harmonisation of Technical Requirements for Pharmaceuticals for Human Use, in case of medical devices: the European Directive on medical devices 93/42/EEC and the ISO Norm 14 155 and ISO 14971, the European Law and the corresponding regulatory Competent Authorities requirements.

#### Patient privacy and confidentiality

The investigators will affirm and uphold the principle of the participant’s right to privacy. Especially, the anonymity of the participants will be guaranteed when presenting the data at scientific meetings or publishing them in scientific journals.

#### Patient and public involvement

This study will be carried out with patient involvement. Specifically, patient advocates and patient associations have been involved in the development of the study design, informed consent and satisfaction questionnaire. For example, within the development of the study design, patient advocates and patient associations highlighted the importance of EUSOBI recommendations for screening in dense breasts. This involvement has been possible since (a) one patient association, Evita Cancro (Portugal), is a partner of the project MammoScreen, (b) the MammoScreen project governance includes a dedicated patient advisory group (PAG), an external board comprising representatives from major national/European patient organisations. PAG is periodically invited to support MammoScreen and provide guidance on clinical protocol, patients’ continuous engagement tools, dissemination and communication strategy.

### Analysis

#### Number of participants

The maximum number of participants will be 10 000 (for all the sites). The sample size was determined using the Buderer approach,^40^ considering that (1) the primary objective of the study is to confirm that MammoWave reaches sensitivity >75% and specificity >90% in BC detection on 10 000 volunteers undergoing breast screening, (2) a two-tailed 95% CI for sensitivity and specificity (CI width=10) and (3) a prevalence of female BC detected due to screening of 0.92%, according to the official English statistics of the National Health Service (NHS) Breast Screening Programme.^41^ A minimum of 8000 participants is required to reach the primary endpoint.

#### Datasets

For the analysis, the following datasets for specific populations have been defined: safety set (SAF), which corresponds to all study volunteers who undergo MammoWave examination; full analysis set (FAS), which includes all volunteers who undergo MammoWave examination and have an evaluable result for the primary endpoint and per-protocol set (PPS), which includes all volunteers who do not have significant CIP violations that regard inclusion/exclusion criteria or that can condition efficacy evaluations.

The primary analyses on both primary and secondary outcomes (except safety evaluation) will be performed on the FAS. The analyses on the PP population will be performed if the PP population differs from the FAS by at least 5% and will be considered supportive of the analyses on the FAS population (sensitivity analysis). The safety and tolerability analyses will be performed on the SAF.

#### Statistical considerations

Data from study participants will be analysed with descriptive statistics (mean, median SD, IQR, min/max for numerical variables, with frequency tables for categorical ones, percentages), 95% CIs as appropriate and graphical analysis on both the entire population and by strata (ie, dense/no dense breast) at the interim analysis and the end of the study. All the methods adopted for the statistical analyses will be described in the statistical analysis plan (SAP), to be issued before the interim analysis. The SAP will include a dedicated section in which the interim analyses are described. We anticipate that, within the interim analysis, sensitivity and specificity will be evaluated. In case the lower limit of the 95% CI is lower than 60% for the sensitivity or 70% for the specificity, the classification algorithm will be updated; otherwise, the study will continue without any modification.

All analyses will be conducted by using SAS (SAS Institute) V.9.4.

#### Data collection, management and monitoring

During each study visit, the study investigator (or designee) will first collect a written informed consent from the volunteer. All clinical data will be collected, anonymised and properly identified with a dedicated study identification number and stored in an eCRF, secured by the Clinical Research Organisation, CRO (JSB Solutions, Italy). The investigator is responsible for ensuring the accuracy, completeness and consistency of the data entered in the eCRF. The data entered in the eCRF (in English language) must be present in the source documents of the volunteers at the investigational centre.

The investigator and the designated person from his/her staff will be trained by CRO for the use of the eCRF and will be responsible for the data entry into the eCRF in accordance with the relevant user guidelines. During the study, the medical records related to the participating volunteer will be kept in the centre. Source data must be available at the site to document the existence of the study participants.

The eCRF system will record the date and time of any entry and/or correction and the user ID of the person making the entry/correction (audit trail). Only authorised investigators will be allowed to view and use the eCRF. The investigators will review all eCRFs and electronically sign and date them for each subject, verifying that the information is complete, true and correct. All fields on the eCRF must be completed as applicable. Volunteers will be provided with a properly designed questionnaire collecting women’s issues and thoughts during the exam with MammoWave, in the language spoken by the women. A copy of all questionnaires will be stored in the subject’s folder as a source document. It is the responsibility of the investigator to correctly enter the data collected by the questionnaire in the relevant sections of the eCRF.

The collection of AEs will start after the time that the informed consent is signed and continue until the volunteer completes the data acquisition. Investigators must obtain all information available to determine the causality and outcome of the AE and to assess whether it meets the criteria for classification as a serious AE requiring immediate notification to the Sponsor. All reported AEs will be documented on source documents and then reported on the eCRF and will include the event description, date of onset date, resolution, seriousness, severity, relatedness, cause, action taken and outcome. The investigator or an authorised designee must assess the causality, severity and relatedness for all the AEs.

Data will not be shared with any outside person or organisation and will not be used except for the development of this study.

The monitoring activities will be conducted by the CRO according to a monitoring plan agreed with the sponsor. The eCRF pages will be reviewed both on-site, by the monitor of the centre, and remotely, by the Data Management staff of the CRO. Data clarification sheets will be generated through the eCRF system, both automatically, through edit checks, and manually, by clinical research associates (CRAs) and/or data managers; the investigator will have to check and solve them. The CRAs will be permitted to inspect all study documentation provided that subject confidentiality is respected; to verify the patients’ informed consent forms, to verify the data reported in eCRF versus the source data and in case of any discrepancies, they will issue the queries. The corresponding investigator is responsible for the review and approval of all query resolutions. The CRAs will be responsible to verify the proper management of study documentation at the site.

## Ethics and dissemination

This study was approved by the Research Ethics Committee of the Liguria Region (CET), Italy, on 13 November 2023 (approval number: CET—Liguria: 524/2023—DB id 13399), the Research Ethics Committee of Complejo Hospitalario de Toledo (CEIC), Spain, on 29 November 2023 (approval number: CEIC—1094), the National Ethics Committee for Clinical Research (CEIC), Portugal on 12 January 2024 (approval number: CEIC—2311KC814), the Bioethical Committee of Pomeranian Medical University in Szczecin, Poland (approval number: KB-006/23/2024) on 13 March 2024 and the Zurich Cantonal Ethics Commission, Switzerland (BASEC 2023-D0101) on 7 June 2024.

The findings of this study will be disseminated through academic and scientific conferences as well as peer-reviewed journals. Such publications will be planned and agreed on with the investigators at each site.

## supplementary material

10.1136/bmjopen-2024-088431 online supplemental file 1
