# Supplementary material for: Microwave imaging for breast cancer screening: protocol for an open, multicentric, interventional, prospective, non-randomised clinical investigation to evaluate cancer detection capabilities of MammoWave system on an asymptomatic population across multiple European countries
Source: BMJ Open. 2024 Nov 2;14(11):e088431. doi: 10.1136/bmjopen-2024-088431 (PMC11535703; doi:10.1136/bmjopen-2024-088431)
Supplement: online supplemental file 1 [file bmjopen-14-11-s001.pdf]

WOMEN'S SATISFACTION QUESTIONNAIRE

ID number: |__|__|__|__|

Clinical center number: |__|__|

Date: |__|__|-|__|__|- |__|__|__|__|
mm dd yyyy

1. Has the examination been interrupted? ☐ **YES** ☐ **NO**

If **YES**, explain why:

____

____

____

2. Did you find the microwave examination tedious?

Please answer the question by indicating your opinion on a scale of 1 to 3

☐ **3** = yes, very much

☐ **2** = a little

☐ **1** = absolutely not

3. Did you find the microwave examination painful?

Please answer the question by indicating your opinion on a scale of 1 to 3

☐ **3** = yes, very much

☐ **2** = a little

☐ **1** = absolutely not

4. Did you find the microwave examination uncomfortable?

Please answer the question by indicating your opinion on a scale of 1 to 3

☐ **3** = yes, very much

☐ **2** = a little

☐ **1** = absolutely not

5. Did you find the examination long-lasting?

Please answer the question by indicating your opinion on a scale of 1 to 3

☐ **3** = yes, very much

☐ **2** = a little

☐ **1** = absolutely not

6. Did you feel adequately informed about the microwave examination?

Please answer the question by indicating your opinion on a scale of 1 to 3

☐ **3** = yes, very much

☐ **2** = a little

☐ **1** = absolutely not

7. Would you recommend the microwave examination? ☐ **YES** ☐ **NO**

If **NO**, explain why:

____

____

____
