# Supplementary material for: Microwave imaging for breast cancer screening: protocol for an open, multicentric, interventional, prospective, non-randomised clinical investigation to evaluate cancer detection capabilities of MammoWave system on an asymptomatic population across multiple European countries
Source: BMJ Open. 2024 Nov 2;14(11):e088431. doi: 10.1136/bmjopen-2024-088431 (PMC11535703; doi:10.1136/bmjopen-2024-088431)
Supplement: Supplementary file 2 [file bmjopen-2024-088431.reviewer_comments.pdf]

PEER REVIEW HISTORY

BMJ Open publishes all reviews undertaken for accepted manuscripts. Reviewers are asked to complete a checklist review form and are provided with free text boxes to elaborate on their assessment. These free text comments are reproduced below.

ARTICLE DETAILS

Title (Provisional)

Microwave imaging for breast cancer screening: protocol for a multicentric, prospective study to evaluate cancer detection capabilities of MammoWave system on an asymptomatic population across multiple European countries

Authors

Álvarez Sánchez-Bayuela, Daniel; Fernández Martín, Juan; Tiberi, Gianluigi; Ghavami, Navid; González, Rubén Giovanetti; Hernánez, Lina Marcela Cruz; Aguilar Angulo, Paul Martín; Darío Martínez Gómez, Aarón; Rodríguez Sánchez, Ana; Bigotti, Alessandra; Khalesi, Banafsheh; Pontoriero, Letizia; Calabrese, Massimo; Tagliafico, Alberto; Castellano, Cristina Romero

---

VERSION 1 - REVIEW

**Reviewer**	**1**
**Name**	**Liang, Lei**
**Affiliation**	**Department of Ultrasound, Aerospace Center Hospital**
**Date**	**07-Jul-2024**
**COI**	**no competing interests**

The manuscript explores the role of the novel MammoWave's AI system for detecting breast cancer, which is innovative. There are some limitations in the manuscript:

1. The article states that the AI classification algorithm used in the MammoWave system is trained based on previous clinical data. However, it does not provide detailed information about the algorithm's validation, sensitivity, and specificity, which are crucial for assessing its performance on new samples.

2. The microwave images from the MammoWave system lack detailed morphological definition, which might affect the interpretation of the images and the accuracy of diagnoses.

3. The article mentions the use of electronic case report forms (eCRFs) for collecting and storing data but does not detail the specific processes for data collection, quality control measures, or how to handle missing data or outliers.

4. The generalizability of the study results may be limited to specific study populations and settings. The size of the breast and its fit to the MammoWave cup size are critical to the imaging results.

5.The article mentions the involvement of patient advocates and patient associations in the development of the study design, informed consent, and satisfaction questionnaire. However, it does not detail how patient feedback specifically influenced the study design.

6. The study mentions an assessment of the safety and tolerability of the MammoWave system but does not elaborate on how adverse events will be monitored and documented or how such events will be managed.

**Reviewer**	**2**
**Name**	**Jones, Lyn**
**Affiliation**	**North Bristol NHS Trust, Bristol Breast Care Centre**
**Date**	**17-Jul-2024**
**COI**	**I declare no competing interests.**

I have the following questions and comments: Abstract: Better clarification of the reference standard in the

abstract. It is currently explained as the output of the conventional breast examination path. It would be

better described as 2 year follow up with histological confirmation of cancer cases. Generally: The

manuscript would benefit from including a clear explanation of the blinding arrangements within the study

(of the intervention (MammoWave) to the current standard conventional imaging (mammogram) and vice

versa). 1. Readers' understanding of the first sentence of the "Hypothesis and Aims" section would be

enhanced by separating the hypothesis from the aim(s) into different sentences. The hypothesis is a

statement that will be proved or disproved by the study. It does not have volition and therefore, it is

incorrect to state that the hypothesis aims to do something. 2. The primary objective should be singular. In

this manuscript there are 3 or 4 objectives stated within the primary objective paragraph. Is the primary

objective to assess the diagnostic accuracy of the intervention (MammoWave) as sensitivity and specificity

at cancer detection? If so, the other stated objectives will need to be added to the list of secondary

objectives. 3. Please could the authors explain why, in a screening study of asymptomatic women, one of

the exclusion criteria is, "women who do not have a mammographic manifestation of the tumour". Is this a

typo? Please explain. 4. When describing the study design, isn't this a paired diagnostic accuracy study

comparing the intervention (MammoWave + AI algorithm interpretation) with the standard (conventional

digital mammography, 2D or tomosynthesis), rather than the current description of "a non-randomised

clinical investigation"? 5. The authors mention an interim analysis once 3,000 volunteers enrolled. How will

this interim data be analysed and what are the potential consequences of the analysis. Will stop/go criteria

be applied? 6. The authors mention that for the reference standard BI-RADS 6 (histologically proven

cancer) is considered as positive and BI-RADS 1,2&3 are considered negative. The explanation for

reference standard BI-RADS 4 and 5 on conventional imaging but no cancer diagnosis on biopsy being

recategorised as BI-RADS 1,2&3 and those with +ve histology being reclassified as BI-RADS 6 needs to be stated in the paper (as it is currently in the trial registration documentation on the Clinical Trials gov

website. 7. Does the team include a qualitative researcher to conduct the embedded

acceptability/satisfaction work and could the questionnaire and plans for structured interview and reporting

be included as supplementary material?

VERSION 1 - AUTHOR RESPONSE

Dear Editor,

Thank you very much for evaluating our manuscript number bmjopen-2024-088431, titled “Microwave imaging for breast cancer screening: protocol for a multicentric, prospective study evaluating MammoWave system”, to be suitable for publication subject to revisions.

In addition to replying to the comments, we have modified the paper according to these comments, with the changes highlighted as tracked-changes in the marked-up copy of your manuscript. We hope that the paper is acceptable for publication in its present form and that it meets the high-quality standard of BMJ Open.

We confirm that neither the manuscript nor any parts of its content are currently under consideration or published in another journal. All authors have approved the manuscript and agree with its submission to BMJ Open.

We look forward to hearing from you soon.

Sincerely,

Gianluigi Tiberi
